# How has the diagnostic approach to parathyroid localization techniques evolved in the past decade? Insights from a single-center experience

**DOI:** 10.1007/s13304-025-02090-8

**Published:** 2025-01-16

**Authors:** Giuseppe Cacciatore, Manuela Mastronardi, Lucia Paiano, Hussein Abdallah, Carmelo Crisafulli, Franca Dore, Stella Bernardi, Nicolò de Manzini, Margherita Sandano, Chiara Dobrinja

**Affiliations:** 1 Division of General Surgery, Department of Medical, Surgical and Health Sciences, University of Trieste, Trieste, Italy; 2 Department of Nuclear Medicine, ASUGI, Trieste University Hospital, Trieste, Italy; 3 SS Endocrinologia, UCO Medicina Clinica ASUGI, Department of Medical, Surgical and Health Sciences, Trieste University Hospital, Trieste, Italy

**Keywords:** Primary hyperparathyroidism, Parathyroid adenoma, 18F-fluorocholine PET/CT, 99mTc-sestamibi scintigraphy, Neck ultrasonography, Preoperative localization, Preoperative imaging techniques

## Abstract

The standardization of preoperative imaging in primary hyperparathyroidism is one of the current challenges of endocrine surgery. A correct localization of the hypersecretory gland by neck ultrasound and 99mTc-sestamibi (MIBI) scintigraphy are not sufficiently sensitive in some cases. In recent years, CT-4D, 18F-Fluorocholine PET/CT, and radio-guided parathyroidectomy have come into common use. The aim of this study is to evaluate the performance of 18F-Fluorocholine PET/CT after prior negative or discordant first-line imaging in patients with primary hyperparathyroidism undergoing parathyroid surgery. Monocentric observational study on patients affected by pHPT undergoing surgery from July 2009 to April 2024 at the Division of General Surgery, Cattinara Teaching Hospital of Trieste. Preoperative, intra-operative, and follow-up data were collected. The imaging methods used were neck ultrasound, 99mTc-sestamibi (MIBI) scintigraphy, and 18F-Fluorocholine PET/CT (since 2018). 172 patients were included. As first radiologic examination, neck ultrasound (US) was performed in 140 cases and 99mTc-sestamibi (MIBI) scintigraphy in 162. Ultrasound and/or scintigraphy imaging were sufficient for the identification of the gland in 127 patients (73.8%), while in 45 patients (26.2%), the localization was defined with other techniques. Particularly, three patients with negative or discordant first-line imaging underwent neck 4D-CT scan who was useful for parathyroid localization all cases (100%). Only one patient received a neck magnetic resonance (MRI) and resulted positive for preoperative localization. Starting in 2018, 29 out of 45 patients underwent 18F-FCH PET/CT yielding a positive result in 29 patients (100%). In other 16 cases (before the introduction of PET/CT in our preoperative imaging study), the preoperative localization was inconclusive and bilateral neck exploration (BNE) was necessary. The sample was homogeneous in terms of age, anthropometric characteristics, and preoperative biochemical parameters. Male/female ratio was 1:5.1. In the intra-operative site, in the cases of exclusive PET/CT positivity, in 28 cases (96.5%), a diagnostic agreement was confirmed, and the gland was macroscopically smaller or normal in size. The combination of ultrasound and MIBI scintigraphy remains the preferred imaging approach for preoperative studies of pHPT. If secondary imaging is required, 18F -FCH PET/CT stands out as the most advantageous option due to its ability to provide anatomical and functional specificity. FCH PET/CT resulted an effective imaging modality with the highest sensitivity of the available imaging techniques for localizing the hyperfunctioning parathyroid gland. Therefore, this method can be recommended in patients showing negative or inconclusive results in the conventional diagnostic imaging.

## Introduction

Primary hyperparathyroidism (pHPT) is one of the most common endocrine disorders and the principal cause of hypercalcemia. This condition can manifest a range of symptoms affecting various systems, including osteoporosis, muscle weakness, nephrolithiasis, hypercalciuria, polyuria, arrhythmia, hypertension, fatigue, depression, and anorexia [1, 2]. The prevalence of pHPT is 1–4 per 1000 in the general population. pHPT is usually diagnosed at an asymptomatic stage as mild hypercalcemia, generally discovered during routine biochemical screening. Open questions still exist in the field of PHPT, including the need to standardize and improve the imaging algorithm for preoperative localization. [1–6]. The diagnosis of pHPT hinges on biochemical assessments, primarily elevated serum calcium and/or PTH levels, while other potential causes of hyperparathyroidism or hypercalcemia must be ruled out [3]. Elevated PTH levels can result from autonomously functioning parathyroid adenomas (up to 85% of cases), diffuse parathyroid hyperplasia (around 15%), or rare parathyroid carcinomas (1–2%) [3–6].

Despite the existence of medical therapies addressing some complications of pHPT, parathyroidectomy remains the sole curative and definitive treatment option [7, 8]. For this reason, it is crucial, from a surgical standpoint, to precisely identify the parathyroid gland requiring removal [9, 10]. Nevertheless, enlarged parathyroid glands are visualized in approximately 80–90% of cases with conventional diagnostic imaging [9, 10].

**Fig. 1:**
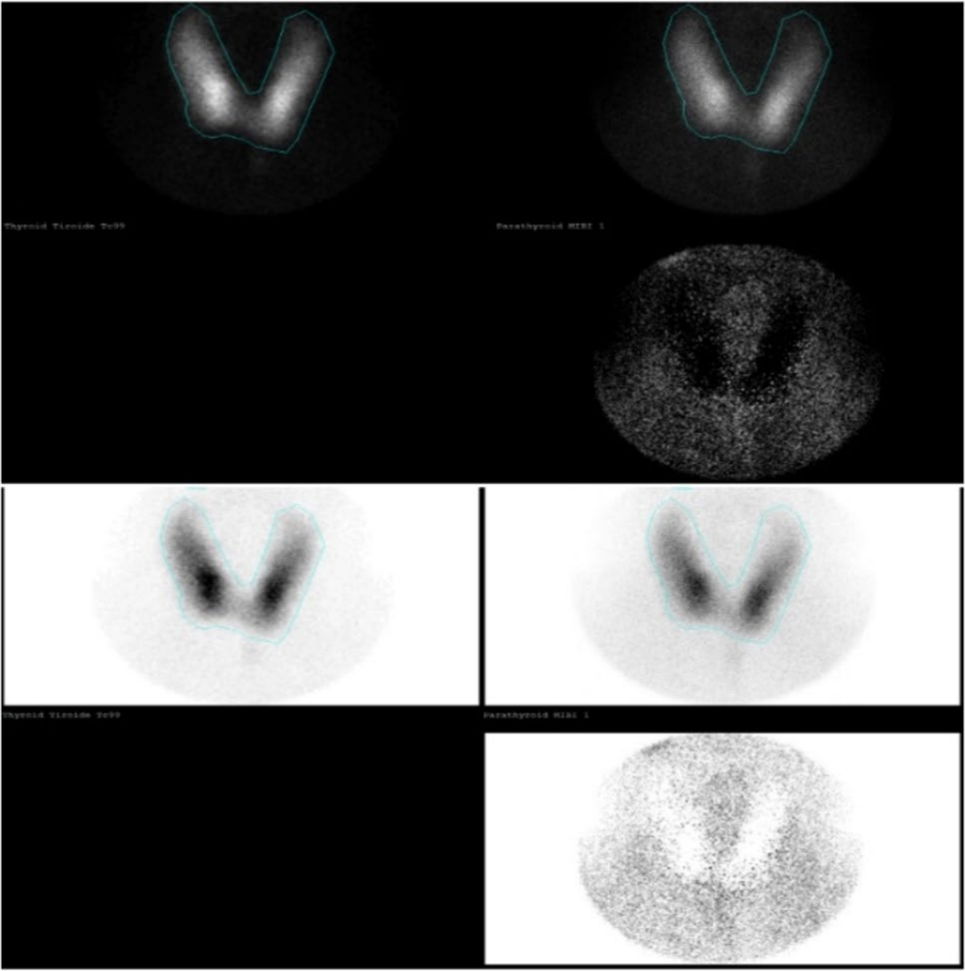
Flow diagram of our patient recruitment process

**Fig. 2:**
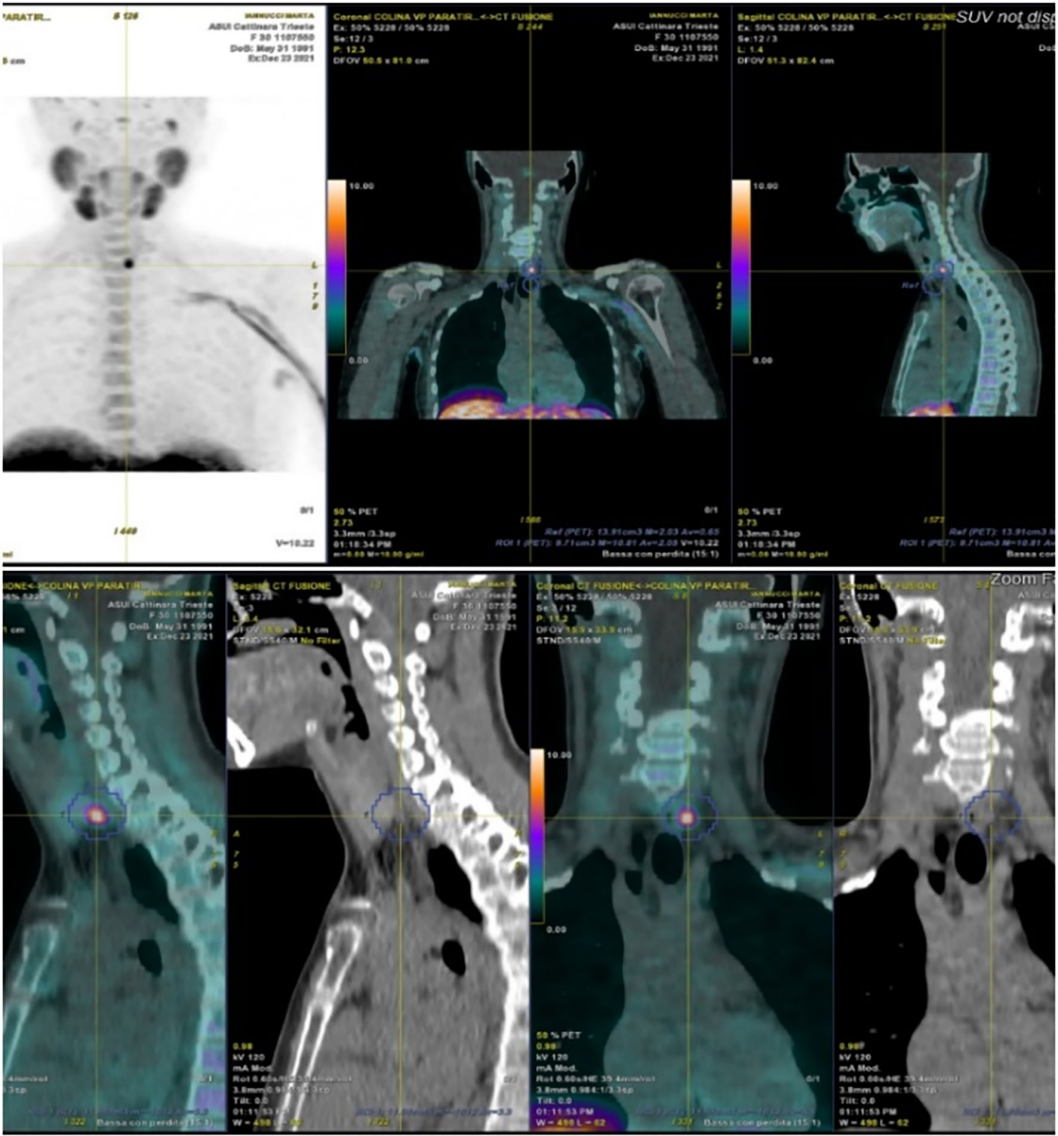
Flow diagram of our results of preoperative imaging and surgery

Today, various imaging techniques are employed in the preoperative evaluation, including neck ultrasound (US), ^99^mTc-sestamibi (MIBI) scintigraphy (Fig. 1), four-dimensional computed tomography (CT), neck magnetic resonance (MRI), and ^18^F-fluorocholine positron emission tomography/computed tomography (^18^F-FCH PET/CT) (Fig. 2) [9]. Neck US and MIBI scintigraphy are the two conventional imaging methods most frequently used for preoperative parathyroid localization, either individually or in combination. Neck US is highly accessible, rapid, and radiation-free, but its sensitivity is somewhat limited, and it may not consistently detect ectopic disease. On the other hand, MIBI scintigraphy excels in localizing adenomas, especially those situated ectopically. However, it may be less effective in pinpointing smaller parathyroid lesions and multiglandular disease, and it involves several hours to complete [11]. CT (4D-CT) is a swift method for identifying small or ectopic parathyroid disease but carries the downside of significant radiation exposure. Consequently, it is often reserved for patients who have previously undergone unsuccessful surgery [3, 10]. Despite its lower cost compared to MIBI scintigraphy, the higher radiation dose may restrict its use, particularly in children and young adults at potentially increased risk of developing thyroid carcinoma [12]. MRI can be valuable for imaging abnormal parathyroids in anatomical areas challenging to explore with US, such as the mediastinum and the tracheoesophageal groove. However, it is typically considered as part of a multimodal approach, not as a standalone method [13].

The advent of hybrid imaging, such as PET/CT, has enabled the co-registration of functional and anatomical information with high spatial resolution [14]. In cases of adenoma or hyperplasia, the upregulation of choline kinase activity leads to enhanced choline uptake. Thus, ^18^F-FCH PET/CT is described as a valuable technique with high accuracy in detecting hyperfunctioning parathyroid tissue in patients with pHPT [15]. This imaging technique offers advantages, including superior spatial tracer resolution, allowing for the detection of even smaller adenomas, and a reduced scanning time due to choline’s rapid kinetics [14]. However, it should be noted that this technique can be relatively costly and may not be readily available at all medical facilities.

The selection of the optimal imaging modality for preoperative studies of pHPT remains a topic of ongoing debate and research. Given this background, our current study aims to discern the most effective imaging approach for visualizing parathyroid glands, providing valuable guidance for surgical intervention in patients with pHPT. Additionally, we present demographic, surgical, and follow-up data from patients referred to our center for pHPT, aiming to contribute to a comprehensive understanding of this multifaceted condition.

## Materials and methods

This is a monocentric observational cohort study on patients affected by pHPT undergoing surgery referred to a tertiary referral center for Endocrine Surgery from July 2009 to April 2024.

We excluded patients with hypercalciuria of renal origin as a cause of increased serum PTH. In addition, we excluded those patients with hypocalciuria for the diagnosis of familial hypocalciuria/hypercalcaemia, and we not included patients with hyperparathyroidism due to genetic causes.

The study was conducted according to strengthening the reporting of observational studies in epidemiology (STROBE) guidelines [16].

The following data have been collected: age, sex, preoperative diagnosis (normocalcemic or hypercalcemic primary hyperparathyroidism), preoperative serum calcium levels, preoperative PTH levels, preoperative phosphorus and vitamin D levels, preoperative phosphaturia, and preoperative urine calcium measurement. Preoperative imaging utilized were neck US, MIBI scintigraphy, neck 4D-CT scan, MRI, and ^18^F-FCH PET/CT (considering that this latter has been in force since October 2018). Type of surgery, operative time in minutes, parathyroid localization at preoperative imaging, intraoperative PTH serum level (ioPTH) before surgical incision (PTH-0), 5 and 15 min after parathyroid retrieval (PTH-5 and PTH-15), histological findings, PTH and calcium serum level at first postoperative day, length of stay (LOS), parathyroid dimensions at histological examination in millimeters (mm), and surgical complication at 30 days were analyzed. Intraoperative PTH (ioPTH) was measured with the quick PTH immunochemiluminometric assay (Access Immunoassay System on Access2 Beckman Coulter, UniCel^®^ DxI 800 Beckman Coulter–Fullerton, CA). A decrease of intraoperative PTH ≥ 50% was defined as sufficient.

Quick parathyroid hormone immunochemiluminometric assay (qPTHa) was performed intraoperatively during 169 (98.2%) surgical procedures. ioPTH was assayed on Access2-Beckman Coulter (Fullerton, CA), on EDTA serial samples [T0: before skin incision, T1: after 5 min and 15 min (T2) from suspected parathyroid adenoma excision]. Surgical procedures are deemed complete when at time T1, a drop of more than 50% of PTH preoperative levels was reached.

**Fig. 3:**
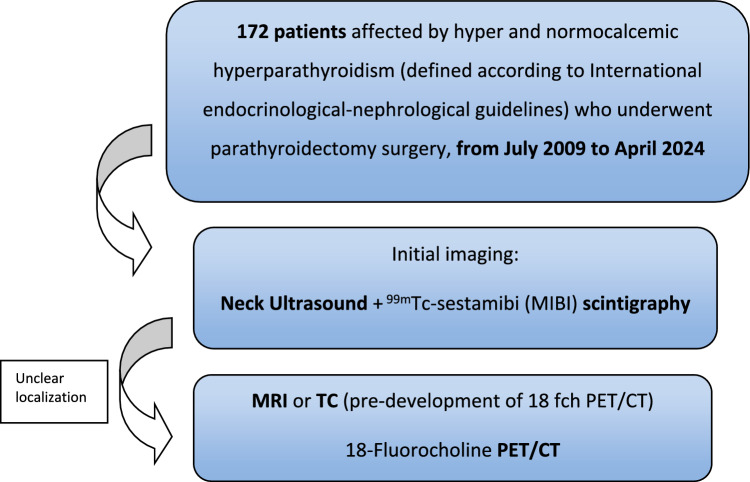
Flowchart illustrating the patient recruitment process

When the PTH levels do not decrease, the remaining parathyroid glands were examined and removed as needed. PTH depletion rate was calculated at 15 min after suspected parathyroid removal as follows: [PTH-0–PTH-15]/PTH-0]*100. Parathyroidectomy was performed by experienced surgeons. The surgical strategy used has been always the same during this long-time frame (2009–2024). When the preoperative imaging was negative, bilateral neck exploration (BNE) was performed (Fig. 3).

A follow-up of 30 days was performed for all patients to assess postoperative complications. Patients were divided according to the preoperative imaging techniques used and compared in terms of demographic, preoperative, intraoperative, and postoperative data. A further analysis was conducted by dividing patients into two groups, according to the presence of a preoperative ^18^F-FCH PET/CT (Group 1—negative or not used 18F-FCH PET/CT; Group 2—positive preoperative ^18^F-FCH PET/CT) and compared in terms of demographic, preoperative, intraoperative, and postoperative data.

### Follow-up

At least a 30-day follow-up was achieved for all patients. Postoperative complications were classified according to Dindo–Clavien Classification [17]. Hypocalcemia was defined as serum calcium < 8.0 mg/dl. In the case of postoperative serum calcium levels lower than 8.0 mg/dl, patients were treated with substitutive therapy with calcium carbonate tablets (1–3 tablets/day) and with a calcitriol (1alpha,25-dihydroxyvitamin D3) 0.5 mg × 2/day.

The serum calcium level of hypocalcemic patients was rechecked every 3 days until normalization of serum calcium and PTH levels.

### Ethical aspects

All data were anonymously collected in a protected electronic database. The study has been conducted according to Good Clinical Practice, to ethical principles from the Helsinki Declaration. The informed consent was signed by every patient enrolled. The study has been registered and approved by the Local Ethical Committee with the following protocol number 346_2024H (GENASUGI/GEN 0036238 P SCRICAQARC 429-P).

### Statistical analysis

Nominal and ordinal variables are expressed as number and percentage; quantitative normal variables as mean ± standard deviation; and quantitative nonnormal variables as median and range. The Shapiro–Wilk test has been used to assess the variable normal distribution. Nominal and ordinal variables are compared with Chi-squared and exact Fisher's tests, quantitative nonnormal with M Mann–Whitney *U* Test when two groups are compared, or Kruskal–Wallis test when more than two groups are compared, normal variables with *T* student test. *P* values ≤ 0.05 were considered statistically significant. Statistical analysis was conducted using SPSS v23.0 (IBM Corp, Armonk, New York, USA).

## Results

A total of 172 patients were enrolled in the study. Demographic and preoperative data are shown in Tables 1, 2, 3. As first radiologic examination, neck ultrasound (US) was performed in 170 cases and 99mTc-sestamibi (MIBI) scintigraphy in 164. Ultrasound and/or scintigraphy imaging were sufficient for the identification of the gland in 141 patients (82%), while in 31 patients (18%), the localization was defined with other techniques. Particularly, two patients with negative or discordant first-line imaging underwent neck 4D-CT scan who was useful for parathyroid localization in 1 case out of 2. Only one patient received a neck magnetic resonance (MRI) and resulted positive for preoperative localization. Since 2018, 29 out of 45 patients underwent 18F-FCH PET/CT yielding a positive result in 29 patients (100%). In other 11 cases (before the introduction of PET/CT in our preoperative imaging study), the preoperative localization was inconclusive and bilateral neck exploration (BNE) was necessary.

**Table 1:** Demographic and preoperative data of 172 patients with primary hyperparathyroidism undergoing surgery

Age (years)	64.1 (19–85)
Sex	
Female	144 (83.7%)
Male	28 (16.3%)
Diagnosis	
Normocalcemic HPT	37 (22.5%)
Hypercalcemic HPT	135 (78.5%)
Preoperative calcemia (g/dl)	11 (9.03–16)
Preoperative PTH (ng/ml)	221.13 (37–3342)
Preoperative ultrasound	
Total	170 (98.8%)
Positive	124/170 (72.9%)
Preoperative MIBI—scintigraphy	
Total	164 (95.3%)
Positive	127/164 (77.4%)
Concordant positive preoperative ultrasound and MIBI—scintigraphy	102 (59.3%)
Preoperative 4D-CT	
Total	3 (1.7%)
Positive	3/3 (100%)
Preoperative MRI	
Total	1 (0.6%)
Positive	0/1 (0)
Preoperative 18F-FCH—PET/CT	
Total	29 (16.8%)
Positive	29/29 (100%)

Nominal and ordinal variables are expressed as number and percentage; and quantitative non-normal variables as median and range

*HPT* hyperparathyroidism; *PTH* parathormone; *MIBI* 99mTc-sestamibi; *4D-CT* four-dimensional computer tomography scan; *MRI* magnetic resonance imaging; *18F-FCH—PET/CT* 18F-fluorocholine positron emission tomography/computer tomography

**Table 2:** Intraoperative PTH data of 169 patients with primary hyperparathyroidism undergoing surgery

Type of surgery	
BNE	15 (8.9)
Open/MIP	150 (88.7)
VAP	4 (2.4)
Operative time (min)	83 (21–205)
PTH-0 (ng/ml)	247.6 (5.4–3800.6)
PTH-5 (ng/ml)	90 (6.8–1120)
PTH-15 (ng/ml)	54.5 (4.5–476.8)

Nominal and ordinal variables are expressed as numbers and percentages; and quantitative non-normal variables as median and range

*BNE* bilateral neck exploration; *MIP* minimally invasive parathyroidectomy; *VAP* video-assisted parathyroidectomy; *PTH* parathormone

**Table 3:** Postoperative and follow-up data of 172 patients with primary hyperparathyroidism undergoing surgery

PTH at day 1 (ng/ml)	36.1 (1–258)
Calcemia at day 1 (g/dl)	9.6 ± 0.78
HE	
Adenoma	113 (77.4)
Atypical adenoma	6 (4.1)
Cancer	6 (4.1)
Hyperplasia	15 (10.3)
Not found	15 (10.3)
LOS (days)	2.9 (1–90)
30-day complication rate (DC)	
I	22 (12.7)
II	2 (1.2)
IIIb	1 (0.6)

Nominal and ordinal variables are expressed as number and percentage; quantitative normal variables as mean ± standard deviation; and quantitative non-normal variables as median and range

*PTH* parathormone; *HE* histological examination; *LOS* length of stay; *DC* Dindo–Clavien classification

The sample was homogeneous in terms of age, anthropometric characteristics, and preoperative biochemical parameters. Male/female ratio was 1:5.1. In the intra-operative site, in cases of exclusive PET/CT positivity, in 28 cases (96.5%), a diagnostic agreement was confirmed, and the gland was macroscopically smaller or normal in size.

Since 2018, 29 patients underwent 18F-FCH PET/CT scans, and all these patients presented with positive results. Among this cohort, two patients faced the challenge of balancing a negative US with a positive MIBI scintigraphy, while two others exhibited the reverse scenario of a positive US with a negative MIBI scintigraphy. An additional patient confronted with a negative US, a negative MIBI scintigraphy, and a positive CT scan.

The analysis was conducted by dividing patients into two groups, according to the presence of a preoperative ^18^F-FCH PET/CT (Group 1—negative or not used preoperative 18F-FCH-PET/CT; Group 2—positive preoperative ^18^F-FCH-PET/CT). The results of the statistical analysis are available in Table 4.

**Table 4:** Demographic, preoperative, intraoperative, and postoperative data of the patients dividing according to the negative or not used (Group 1) or positive (Group 2) preoperative 18F-FCH PET/CT

	GROUP 1 Pet no 143	GROUP 2 Pet yes 29	*P* value
Sex	117 F (81.8%) 26 M (18.2%)	27 F (93.1%) 2 M (6.9%)	0.173
Preoperative diagnosis	32 (33.4%) normocalcemic 111 (77.6%) hypercalcemic	5 17.2% normocalcemic 24 82.8% hypercalcemic	0.628
US yes	141 (98.6%)	29 (100%)	1.00
US no	2 (1.48%)	0	
US positive	118 (82.5%)	6 (20.7%)	*P* < 0.001
US negative	25 (17.5%)	23 (79.3%)	*P* < 0.001
Scintigraphy yes	135 (94.4%)	29 (100%)	
Scintigraphy no	8 (5.6%)	0	*P* 0.354
Scintigraphy positive	123 (86%)	4 (23.8%)	
Scintigraphy negative	20 (14%)	25 86.2%	*P* < 0.001
Negative US and MIBI scintigraphy	2 (1.4%)	19 (65.5%)	
Type of surgery			*P* 0.849
ECB	11 (7.7%)	4 (13.8%)	
Focused parathyroidectomy	126 (88.1%)	24 (82.8%)	
Parathyroidectomy + thyroid surgery	5 (3.5%)	1 (3.4%)	
Parathyroidectomy + thyroid lobectomy + central lymph-node dissection	1 (0.7%)	0	
Immediate postoperative complications			*P* = N.S
Laryngeal nerve palsy (transient)	10 (7%)	2 (6.9%)	
Hypocalcemia (11 transient + 1 permanent)	8 (5.6)	4 (13.8%)	
Complications 30 days Clavien–Dindo			*P* 0.055
1	20 (14%)	2 (6.9%)	
2	1 (0.7%)	1 (3.4%)	
3	0	1 (3.4%)	
Duration of surgery in minutes	Median 59 min (min 30 max 60)	Median 62 min (min 38 max 145)	*P* 0.221
Parathyroid dimeter in millimeter	Mean 15 (min 10 max 27)	Mean 10 (min 7 max 27)	*P* 0.151
Reintervention of parathyroidectomy	2 (1.4%)	1 (3.4%)	

Nominal and ordinal variables are expressed as number and percentage; quantitative normal variables as mean ± standard deviation; and quantitative non-normal variables as median and range

*M* male, *F* female, *HPT* hyperparathyroidism; *PTH* parathormone; *MIP* minimally invasive parathyroidectomy; *VAP* video-assisted parathyroidectomy; *LOS* length of stay; *DC* Dindo–Clavien classification; *ng/ml* nanograms/milliliters

Almost all patients with hyperparathyroidism presented with hypercalcemia at preoperative diagnosis. Almost all patients performed preoperative US and MIBI scintigraphy for both group 1 and group 2. Ultrasound used as first-level test examination in the preoperative diagnosis resulted positive in 82.5% in group 1 and 20.7% in group 2 with statistical significance (*P* < 0.001). Instead, ultrasound resulted negative in 17.5% in group 1 and in 79.3% in group 2 with statistical significance (*P* < 0.001). MIBI scintigraphy used as first-level test examination in the preoperative diagnosis resulted negative in 14% in group 1 and 86.2% in group 2 with statistical significance (*P* < 0.001) and MIBI scintigraphy positive evidence of 86% in group 1 and 23.8% in group 2. Assessing the proficiency of each technique in pinpointing the parathyroid and its alignment with the surgical findings, according to guidelines [18], the US correctly identified the lesion in 124 cases out of 172 (72.1%); MIBI scintigraphy in 127 out of 172 (73.8%); ^18^F-FCH PET/CT in 29 cases out of 29 (100%); 4D-CT in 1 out of 2 cases (50%); and MRI in 1 case out of one (100%). In 102 (59.3%) patients, there were a concordant positive preoperative ultrasound and MIBI scintigraphy, while a negative US and MIBI scintigraphy evidence there was in 2 cases (1.4%) in group 1 and 19 cases (65.5%) in group 2: this demonstrates the ^18^F-FCH-PET/CT’s importance for preoperative diagnosis. Comparing the different imaging techniques, in 102 cases out of 172 (59.3%), there was a concordance between US and MIBI scintigraphy. US and ^18^F-FCH PET/CT were concordant in 6 cases out of 29 (20.1%), as ^18^F-FCH PET/CT and MIBI scintigraphy. US and 4D-CT were concordant in 1 case out of 2 (50%), as MIBI scintigraphy and 4D-CT. In the patient that underwent MRI, this technique was discordant with both US and MIBI scintigraphy.

Regarding the type of surgery, in group 1, 11 patients (7.7%) underwent ECB, while in group 2, 4 patients (13.8%). 126 patients (88.1%) in group 1 and 24 patients (82.8%) in group 2, underwent focused paratyroidectomy. In 5 cases for group 1 and in 1 case for group 2, parathyroidectomy has been associated with thyroid surgery. Only in one case of group 1 was a patient subjected to parathyroidectomy, thyroid lobectomy, and central lymph-node dissection.

Regarding the ioPTH results, quick parathyroid hormone immunochemiluminometric assay (qPTHa) was performed intraoperatively during 169 (98.2%) surgical procedures. The results are summarized in Table 2. Blood drawings were routinely performed before skin incision (T0), 5 min (T1), and 15 min (T2) after the excision of suspected parathyroid adenoma. Surgery procedures were concluded when at T1 has been reached a drop in PTHIO levels > 50% with respect to preoperative levels.

In 159 patients (94.1%), a drop in ioPTH value was more than 50% at T1; for 10 (5.9%), the drop was 19.5% (4 patients where the parathyroid adenoma does not find and 6 parathyroid hyperplasia).

Among the 172 patients, 17 experienced a prolonged LOS exceeding 4 days. The extended LOS was primarily attributed to concomitant medical conditions, such as pancreatitis in one case and pre-existing chronic conditions (cardiovascular, pulmonary, or renal) that necessitated nursing home care for 11 patients. Two cases involved extended LOS due to postoperative complications. Overall, postoperative complications were encountered in 25 patients, accounting for 14.5% of cases. These complications were primarily categorized as grade I (12.7%), grade II (1.2%), and one patient (0.6%) required reintervention for postoperative bleeding (grade IIIb). This latter patient had an inferior left parathyroid extending into the mediastinum. In addition, 12 patients developed transient laryngeal nerve paralysis. No permanent laryngeal nerve paralysis was observed. Transient hypocalcemia was noticed in 12 patients: one of which progressed to permanent hypocalcemia (i.e., persistence more than 6 months after surgery). Among the transient unilateral recurrent paralysis, we specify that there were four cases where postoperative laryngoscopy has shown a slowing of vocal cord motility without clinical evidence of dysphonia with recovery within 14 days after surgery.

The median follow-up duration extended to 10 months, with a range spanning from 1 to 117 months. Only one patient required calcium therapy for 6 months post-surgery, indicative of permanent hypoparathyroidism. No recurrent laryngeal nerve lesions were reported throughout the study.

Three patients (1.7%) developed a recurrence during the long-term follow-up: two of these were found with 99mTc-sestamibi (MIBI) scintigraphy, while the third was found with ^18^F-FCH PET/CT.

## Discussion

The present study offers insights into a 16-year surgical experience with pHPT analyzing the preoperative tests for biochemical diagnosis and the techniques utilized for parathyroid localization before surgery. In our comprehensive case series, preoperative imaging demonstrated its pivotal role, with US emerging as the most frequently utilized modality (employed in 98% of patients). Before the advent of ^18^F-FCH PET/CT, our clinic predominantly relied on MIBI scintigraphy as the second-level imaging modality. However, this approach proved to be less reliable, yielding negative results in 17.5% of cases (false-negative results), which posed challenges in surgical planning.

In our case series, a total of 21 patients presented a negative US and MIBI scintigraphy (2 in Group 1 and 19 in Group 2). In one case, a 4D-CT scan was performed and confirmed the presence of an adenoma; in 19 cases, a ^18^F-FCH PET/CT confirmed the presence of an adenoma, but 1 patient underwent surgery with negative US and MIBI scintigraphy. In the latter case, parathyroid cancer was found. In addition, the positivity of ultrasound as the first diagnostic level was higher in the first group compared to the second group, respectively, 82.5% and 20.7% with statistical significance (*P* < 0.001). Similarly, the negativity in both ultrasound and MIBI scintigraphy was higher in the second group compared with the first group, respectively, 79.3% and 86.25%, with statistical significance (*P* < 0.001). This points out the importance of 18 F-FCH PET/CT positivity in unclear cases when ultrasound and/or MIBI scintigraphy were negative. These findings could lead to several considerations. First of all, localizing parathyroid glands can be challenging. These discrepancies might stem from various factors, such as the size of the glands, their location within the neck, or variations in individual anatomy. Furthermore, in case of conflicting or inconclusive results from imaging, surgeons potentially have more difficulties in locating and removing the affected parathyroid glands. Therefore, alternative or more advanced imaging methods may be considered.

The introduction of ^18^F-FCH PET/CT marked a significant turning point in our approach. This advanced imaging technique consistently confirmed the preoperative diagnosis in all cases, enabling us to precisely identify the parathyroid glands targeted for removal [19, 20]. Remarkably, we were able to locate the parathyroid in 91.3% of cases directly, with only 8.7% classified as having BNE. In terms of postoperative outcomes, our study reported a 14.5% complication rate, excluding BNE. There were three cases necessitating reintervention and another that required calcium therapy at the end of the 30-day follow-up, with persistent hypoparathyroidism at 6 months after surgery. These findings emphasize the importance of accurate preoperative imaging in optimizing the management of pHPT and minimizing postoperative complications. No differences were found in terms of intraoperative and postoperative data when the population was divided according to the preoperative imaging or according to the presence of a preoperative ^18^F-FCH PET/CT.

Regarding the three cases reoperated for parathyroidectomy, there were 2 cases in group 1 (without PET/CT) and one in group 2 (with PET/CT). The patients were reoperated respectively after 5 and 4 years after the first intervention and were studied by a MIBI scintigraphy (group 1) and on the contrary the last patient (group 2) was reoperated after 2 years and a parathyroidectomy was performed, discovered by PET/CT.

As highlighted in the existing literature, the ability to accurately localize the affected parathyroid gland prior to surgery has far-reaching benefits. It not only enhances the planning of the surgical approach but also increases the feasibility of performing minimally invasive surgery, which in turn reduces postoperative pain, shortens the LOS, and ultimately leads to improved esthetic outcomes [21]. Targeted surgery, as opposed to the traditional BNE, not only achieves better overall results but also minimizes morbidity.

The combination of US and MIBI scintigraphy has been a well-established approach, with the potential to attain a sensitivity ranging from 80 to 90% [22]. In our cohort of patients, the combined use of US and MIBI scintigraphy proved sufficient for the localization of the hypersecretory gland in a commendable 76.7% of cases. However, the most remarkable results were observed when utilizing ^18^F-FCH PET/CT, as it facilitated the identification of the abnormal gland in 100% of cases. Impressively, in 93.3% (14 out of 15) of patients with positive ^18^F-FCH PET/CT results, MIP became a feasible option. Only one patient, who underwent ^18^F-FCH PET/CT, necessitated BNE.

The reason for the exceptional success of ^18^F-FCH PET/CT lies in its superior spatial resolution of the tracer, which enables the detection of even smaller adenomas. This enhanced resolution is a result of the rapid kinetics of choline, which proves particularly advantageous in identifying and localizing these abnormal parathyroid glands [14]. These findings underscore the significant advancement that ^18^F-FCH PET/CT represents in preoperative imaging for primary hyperparathyroidism, with the potential to revolutionize surgical planning and outcomes. Considering our experience, we propose the flow diagram in Fig. 3. ^18^F–FCH PET/CT can be useful also when US and MIBI scintigraphy are both positive but in different locations [23, 24].

Within our patient cohort, the pathological findings revealed a diverse spectrum of parathyroid conditions. Most cases consisted of parathyroid adenomas, accounting for 131 cases or 76.2% of the total patients. Atypical adenomas were identified in 6 patients, representing 3.5% of the cohort. Additionally, 16 cases (9.3%) were characterized by parathyroid hyperplasia, and another 5 cases (2.9%) were determined to be parathyroid cancers. These findings are consistent with the current body of literature, further validating the prevalence of these parathyroid pathologies [5, 25]. Intriguingly, in 6 cases (3.5%), the initially suspected parathyroid adenoma was not found during the surgical procedure. In one case, the ^18^F-FCH PET/CT revealed the presence of an adenoma. However, in other cases, this imaging was not conducted as it was not available in our facility at that time. Given that the patients had pHPT, the potential utilization of this examination could have offered an opportunity for a more comprehensive diagnostic approach, potentially leading to improved diagnosis and management.

Regarding the population included in the study and considering the preoperative diagnosis, in our series, about 22.5% of patients presented a normocalcemic primary hyperparathyroidism (nPHPT). This condition is not well understood in medical literature. Normocalcemic hyperparathyroidism presents a unique diagnostic challenge due to its normal serum calcium levels despite elevated parathyroid hormone levels. nPHPT can overlap with primary or secondary hyperparathyroidism, necessitating thorough evaluation to differentiate between these conditions. The diagnosis of nPHPT requires excluding secondary causes of elevated parathyroid hormone through a comprehensive clinical history, physical examination, and targeted laboratory investigations. Treatment strategies for nPHPT focus on addressing the underlying parathyroid gland abnormalities, surgical parathyroidectomy in select patients, and long-term monitoring to assess treatment efficacy and prevent the recurrence of hyperparathyroidism-related complications [26–29].

It is important to acknowledge certain limitations of our study, primarily its retrospective design, which may introduce inherent biases. Therefore, prospective studies specifically designed to investigate the localization of parathyroid glands could provide more robust and reliable evidence. Additionally, not all patients had the opportunity to undergo ^18^F-FCH PET/CT, as this modality was introduced later in our clinical practice. However, it is worth noting that the study's strengths lie in the extensive 16-year study period, allowing for a comprehensive analysis of the cases, and the fact that all surgeries were performed by experienced endocrine surgeons. These factors contribute to the robustness and reliability of the data, underscoring the clinical significance of the findings in the context of managing primary hyperparathyroidism.

Moreover, the success of minimally invasive surgery in managing pHPT critically hinges on the precise preoperative localization of the overactive parathyroid gland. For patients initially presenting with negative preoperative imaging, ^18^F-FCH PET/CT scans have emerged as a valuable diagnostic tool. These scans have demonstrated their ability to reveal even small parathyroid adenomas, even in cases where patients may exhibit normal calcium levels or hyperparathyroidism with normal parathormone levels, rendering ioPTH measurement less informative.

In our series, the use of the ioPTH assay enables the effectiveness of parathyroidectomy operations as there is a connecting factor between hormone kinetics during the operation and the removal of pathological parathyroid gland. A reduction in the ioPTH serum levels of over 50% with respect to the basal swab is considered a significant drop and is a confirmation of the adequacy of the surgical treatment.

The recent literature underscores the effectiveness and high accuracy of ^18^F-FCH PET/CT scans as a preoperative localization method for parathyroid adenomas, positioning it as a viable alternative to conventional imaging techniques [9, 10, 14]. This advanced technology offers superior spatial resolution, shorter scanning times, and reduced radiation exposure compared to methods like MIBI scintigraphy and 4D-CT, consistently delivering detection rates exceeding 90% [30]. It is important to note that ^18^F-FCH PET/CT is typically not recommended as the first-line imaging modality but rather reserved for patients with negative or inconclusive initial imaging results [9, 10, 14, 31]. In the future, it is conceivable that ^18^F-FCH PET/CT scans could become integrated into the first-level imaging for pHPT diagnosis, given their exceptional performance. Since the introduction of ^18^F-FCH PET/CT scans in our clinical practice, we have observed a notable increase in the number of hyperparathyroidism patients scheduled for surgery. Beyond providing a biochemical diagnosis, this technique offers the crucial advantage of precisely localizing the presumed adenoma. Nevertheless, it is essential to recognize that due to its remarkable accuracy, and ^18^F-FCH PET/CT can identify even the tiniest adenomas that may not be visually apparent during surgery. Consequently, in cases where ioPTH cannot be employed or in situations of pHPT with normal PTH levels, frozen section analysis stands as a reliable and complementary confirmation method [32]. This multimodal approach ensures the best possible outcomes in the management of pHPT, emphasizing the importance of ongoing advancements in diagnostic tools and surgical techniques.

## Conclusions

This study confirms the enormous importance and effectiveness of 18 F-FCH PET/CT in particular in unclear cases where ultrasound and/or MIBI scintigraphy are negative. In these patients, 18 F-FCH PET/CT can be used as an additional weapon in preoperative diagnostic management. US in combination with MIBI scintigraphy remains a widely employed imaging approach for the preoperative evaluation of pHPT. However, in situations where a more comprehensive investigation is warranted, ^18^F-FCH PET/CT emerges as a powerful and advantageous alternative. The strength of ^18^F-FCH PET/CT lies in its ability to integrate anatomical and functional specificity, a feature that significantly enhances its diagnostic utility. While this advanced imaging approach undeniably offers numerous benefits, including precise localization of pathological parathyroid glands, it is important to acknowledge its cost and limited availability in some healthcare settings. Nonetheless, its use can effectively prevent unnecessary surgical procedures by providing an accurate roadmap to the specific parathyroid lesion, thus ensuring focused and highly successful surgical interventions, leading to a noteworthy advancement in the field of endocrine surgery. However, further comprehensive studies are needed to confirm our data.

## Data Availability

Data are available under specific request.
